# The Impact of COVID-19 Social Isolation on Physical Activity and Sedentary Behavior in Brazilian Children and Adolescents

**DOI:** 10.1055/s-0044-1800941

**Published:** 2025-03-12

**Authors:** Fernando Leite Miranda, Carlos Henrique Fernandes, Lia Myiamoto Meirelles, Flavio Faloppa, Benno Ejnisman, Moises Cohen

**Affiliations:** 1 Programa de Pós-Graduação em Ciências da Saúde Aplicadas ao Esporte e à Atividade Física, Departamento de Ortopedia, Escola Paulista de Medicina, Universidade Federal de São Paulo (UNIFESP), São Paulo, SP, Brasil

**Keywords:** adolescent, children, COVID-19, exercise, surveys and questionnaires

## Abstract

**Objective** To measure the impact of social isolation on physical activities and sedentary behavior in Brazilian children and adolescents. To assess whether factors such as age, gender, days of week, type of housing, and the population of the city also had an impact.

**Methods** The study was approved by the Medical Ethics Committee, and consent from parents was obtained. A Google Form (Google LLC, Mountain View, CA, USA) was sent to parents by email and WhatsApp (Meta Platforms Inc., Menlo Park, CA, USA). Parents answered the C-PAQ.PT questions for two different periods of time, one month before and during social isolation.

**Results** There was a statistically significant reduction in the mean physical activity ( *p* < 0.001) and an increase in the mean sedentary activities ( *p* < 0.001) on weekdays and weekends during social isolation. There was a greater reduction in basketball, handball, and running in the 10 to 14-year-old group, as well as playing in the playground in the 5-to-9-year-old group. There was a statistically significant decrease in the practice of tag and cycling, respectively, among females and males. We did not observe statistically significant differences between types of housing and the number of inhabitants in the city.

**Conclusion** The C-PAQ.PT allowed a quantitative assessment to identify the variations in physical activities and sedentary behaviors during social isolation. We have observed that only two physical activities were impacted by gender. The changes were not influenced by the number of inhabitants in the city or the type of housing.

## Introduction

On January 30, 2020, and March 11, 2020, respectively, the World Health Organization declared the coronavirus disease 2019 (COVID-19) infection a global public health emergency and pandemic. Many countries have implemented a series of control measures to prepare for and respond to COVID-19. To prevent the disease from spreading, complete or partial lockdowns were instituted. Social measures, including movement restrictions, the closure of schools and businesses, social isolation, and international travel restrictions, were implemented to prevent the introduction and limit the movement of the virus. 1

One way to assess the level and frequency of physical activity in children outside the school environment is through the use of accelerometers, which act as movement sensors. This is a method that allows you to collect information immediately, at low cost, and with great applicability, considering different contexts and practices of physical activity. 2 In a situation of social isolation, the use of an accelerometer for this research was practically impossible. For this reason, we conducted a literature search to identify alternative methods that could enable the evaluation of the degree and frequency of physical activity among children outside the school setting without compromising social isolation. Self-report questionnaires may still be the only feasible way to assess physical activity in many situations and for aspects that are not easily measured objectively. 3 Questionnaires have been proposed for different age groups and have been validated by comparing questionnaires with the concomitant use of accelerometers. 4 5 6

The proliferation of web-based epidemiological studies employing online recruitment techniques is increasing; however, the optimal approach to maximize recruitment rates remains uncertain. 7

Although there are studies performed in Brazil 8 and systematic reviews, 9 10 there is a lack of quantitative assessments of the impact of social distancing on physical activity and sedentary behavior in children. The primary objective of this study was to identify and quantify whether physical activities and sedentary behavior underwent modifications in comparison to pre-pandemic levels in children and adolescents during the period of social isolation. Secondary objectives were to investigate whether changes were influenced by age, gender, type of day of the week or weekend, housing (vertical or horizontal), and the number of inhabitants in the city.

## Materials and Methods

The cross-sectional observational study was approved by the Medical Ethics Committee under number CAAE: 31168920.6.0000.5505. It required the participation of volunteers, such as parents or guardians, who had personal relationships with the researchers and answered the questions. An informed consent form was included prior to the questionnaire to inform all parents or guardians that they would consent to participate in the research by clicking on an “I want to participate” item. We included answers from parents or guardians who live with children of both genders, aged from 5 to 14 years old, and who live in a city and have experienced social isolation due to the COVID-19 pandemic. We included answers from parents or guardians who live with children of both genders. Questionnaires' answers were excluded if repeated.

### Questionnaire

We used Google Forms (Google LLC., Mountain View, CA, USA) to specifically develop a questionnaire for the purpose of this investigation. We have employed a translation and cross-cultural adaptation of the children's physical activity questionnaire (C-PAQ) 11 12 to Portuguese. 13 This included 49 questions regarding physical activities and sedentary behaviors. The answers were straightforward and could be completed within a few minutes.

There were two situations where the parents or guardians were asked to participate. The first was based on the activities performed by children and adolescents about a month before social isolation. The second was based on the activities performed by children and adolescents upon receiving the form during social isolation. In addition to the questionnaire's answers, we also requested the children's identification data, the city of residence, and the type of housing. The survey was distributed to 350 parents or guardians through electronic mail and WhatsApp (Meta Platforms Inc., Menlo Park, CA, USA) between June 1 ^st^ , 2020, and June 15, 2020.

To perform the analysis, the children were divided into two types of housing: apartment (vertical) or house (horizontal) and cities with more than or less than 500,000 inhabitants. The age range was chosen because it is the most common among Brazilian elementary and middle school students, and was divided into two age groups: 5 to 9 and 10 to 14-years-old.

### Statistical Methodology

We used the Wilcoxon test to evaluate the differences between before and during the lockdown. The chi-squared test was used to determine whether or not the variables were dependent or associated with each other. Each comparison was associated with a *p* -value. For this statistical analysis, the following were used: the IBM SPSS Statistics for Windows (IBM Corp., Armonk, NY, USA) version 20.0, Minitab 16 (Minitab Inc., State College, PA, USA), and the Microsoft Excel 2010 (Microsoft Corp., Redmond, WA, USA).

## Results

Among the 171 completed forms, 35 were excluded due to a lack of information that prevented data analysis. In total, 136 forms were analyzed. Regarding age, the groups were aged from 5 to 9 and 10 to 14-years-old, being comprised of 70 and 66 children, respectively. The groups of those living in apartments and houses were comprised of 52 and 84 children, respectively. Regarding gender, 66 children were female, 65 were male, and 5 were not identified by their parents. We received questionnaires from residents of 27 cities, distributed across eight states of Brazil. Regarding the size of the city, 102 children lived in cities with over 500,000 inhabitants, 32 with less than 500,000 inhabitants, and two children did not inform where they lived.

**Fig. 1:**
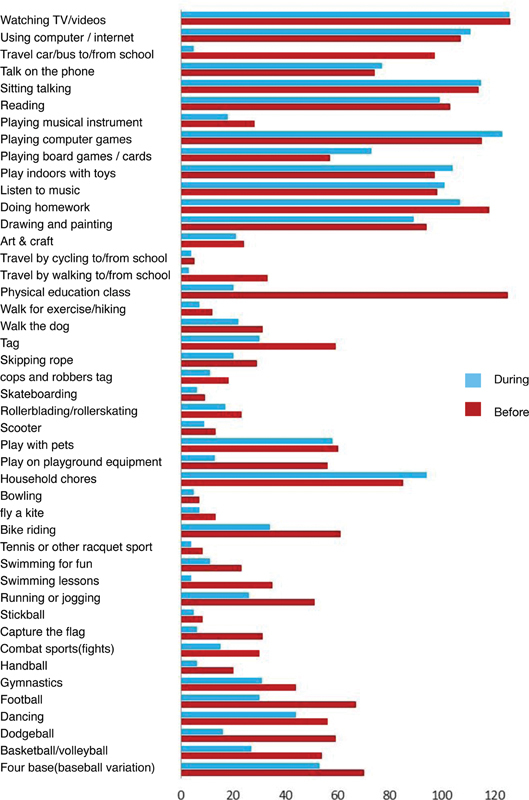
The content of the graph depicts the number of children and adolescents engaged in each activity prior to and during social isolation.

**Fig. 2:**
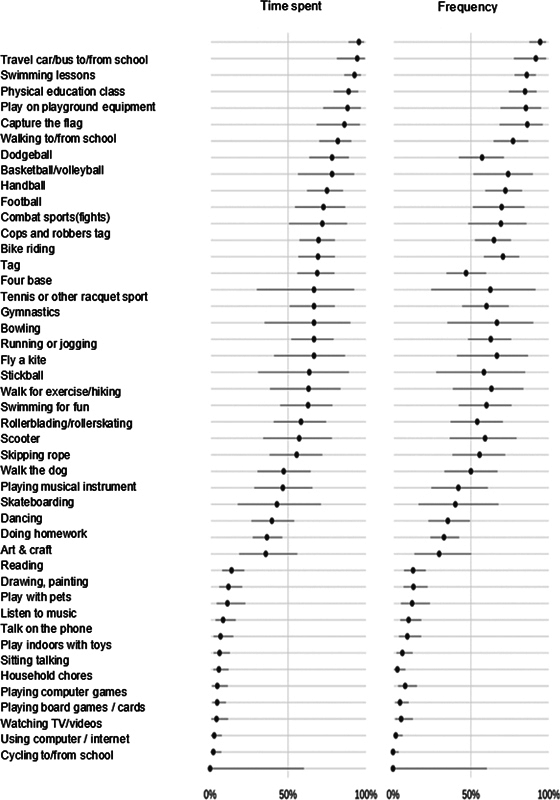
The contents of the spreadsheet indicate the reduction rate of time spent and frequency of each activity.

**Fig. 3:**
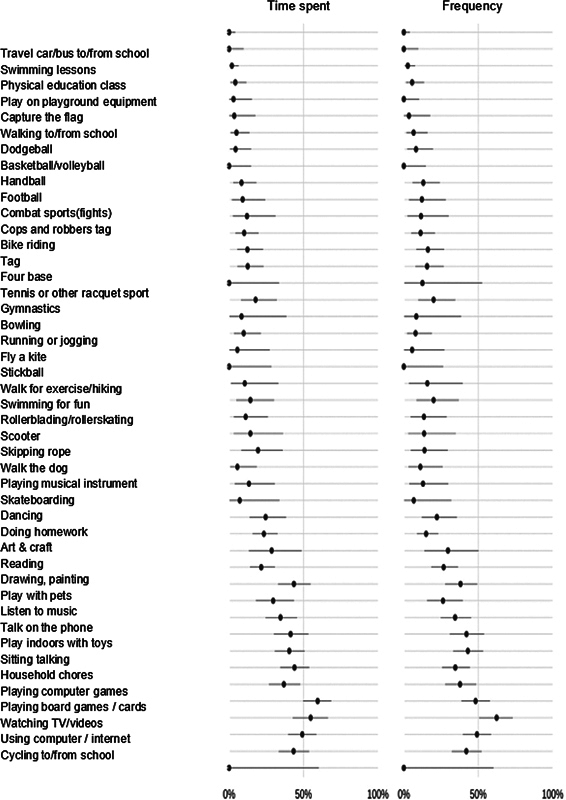
The contents of the spreadsheet indicate an increase in the rate of time spent and frequency of each activity.

Fig. 1 shows the 45 activities that had a variation in the number of children and adolescents upon comparing before and during social isolation. Of which 17 and 28 were considered physical and sedentary activities, respectively. There was a significant reduction in the number of practitioners, total time, and frequency of activities such as basketball, dodgeball, soccer, handball, fights, capture-the-flag, running, swimming lessons, swimming for fun, riding a bicycle, playing in the playground, tag, physical education class, walking to school, and going by car or bus to school ( Fig. 2 ). Activities that were performed indoors had a significant increase in total time and frequency despite lacking a significant increase in the number of participants in drawing and painting activities, playing with toys indoors, board/card games, games on electronic devices, sitting and chatting, browsing the internet, and watching videos/television ( Fig. 3 ). Some activities were practiced only by a few children, which resulted in a high degree of uncertainty in the estimates.

The frequency and duration of each activity were categorized to determine the number of minutes each activity was practiced throughout the weekdays and weekends, before and during the pandemic. The categorization of his behavior was based on the average frequency (number of times per week) and average intensity (period), as shown in Table 1 . We obtained the mean time practiced by participants in all activities by multiplying frequency by intensity. Therefore, the time spent practicing activities before and during the pandemic on weekdays and weekends was compared. For this analysis, we will only consider responses from participants who cited practice both before and during the pandemic. Thus, we have the condition of paired data, that is, when the same participant is the researcher and controls themselves.

**Table 1:** The behaviors were categorized based on frequency (number of times per week) and intensity (period)

Frequency	Categorization
1–2 times	1.5
3–4 times	3.5
5 or more times	5
**Intensity**	
1–30 minutes	15
31–60 minutes	45
61–180 minutes	120
More than 180 minutes	240

During periods of social isolation, there was a reduction in the duration of physical activity and a rise in sedentary behaviors, both on weekdays and weekends. During weekdays, the mean physical activity before and during social isolation was 197.0 and 153.4 minutes, respectively ( *p* < 0.001). On weekends, the average physical activity before and during social isolation was 192.1 and 149.4 minutes, respectively ( *p* < 0.001). On weekdays, the mean sedentary activities before and during social isolation were 268.1 and 393.3 minutes, respectively ( *p* < 0.001); on weekend days, they were 251.8 and 346.2 minutes, respectively ( *p* < 0.001), as shown in Table 2 .

**Table 2:** A comparison of the duration of physical activity prior to and during the pandemic (Wilcoxon test)

Social isolation			Mean	Median	SD	Q1	Q3	N	CI	*p* -value
Physical activity	Week	Before	197.0	67.5	262.4	22.5	180.0	273	31.1	< 0,001
		During	153.4	52.5	270.5	22.5	157.5	273	32.1	
	Weekend	Before	192.1	67.5	304.9	22.5	180.0	177	44.9	0,019
		During	149.9	22.5	282.2	22.5	157.5	177	41.6	
Sedentarism	Week	Before	268.1	157.5	352.5	22.5	420.0	1,378	18.6	< 0,001
		During	393.3	157.5	414.6	52.5	600.0	1,378	21.9	
	Weekend	Before	251.8	67.5	354.4	22.5	420.0	1,143	20.5	< 0,001
		During	346.2	157.5	412.6	22.5	600.0	1,143	23.9	

**Abbreviations:** CI, confidence interval; Q1, first quartile; Q3, third quartile; SD, standard deviation.

During social isolation, there was a reduction in activities that can be performed outdoors in groups or related to school activities and an increase in the frequency and amount of participation in sedentary activities that were performed indoors. In the 10 to 14-year-old group, there was a greater reduction in basketball, handball, and running; whereas in the 5 to 9-year-old group, there was a greater reduction in playing in the playground. There was a statistically significant decrease in tag and cycling practices among females and males, respectively. In most activities, there were no statistically significant differences between different types of housing. As for the frequency, only the activities of playing with toys indoors and watching television/videos had a difference between the groups. Although the difference in the reduction of these activities was statistically significant, their values are small, and the activities had relatively little reduction in the sample as a whole. The number of inhabitants of the city had no statistical significance, meaning that it did not influence changes in the activities of children and adolescents.

## Discussion

The diminution in the frequency and intensity of physical and sedentary activities among children during social isolation resulting from the COVID-19 pandemic may have an impact on the habit of sustaining a healthy life, including the postural balance in children. 14

Self-reported questionnaires were used on a large scale to evaluate and monitor physical activity in children and adolescents. The C-PAQ is a tool that can be downloaded from the website ( https://www.mrc-epid.cam.ac.uk/wp-content/uploads/2014/08/CPAQ.pdf ), and the questionnaire was previously validated. 12 Due to its characteristics, we considered it a good tool to evaluate the impact of social isolation due to the COVID-19 pandemic on the activities of children and adolescents living in Brazil. The translation and cultural adaptation of a self-assessment questionnaire enabled the use of instruments to compare groups with different languages and cultures, which is very useful in clinical practice. 15 16 17 The eight activities exchanged for Brazilian culture showed a high response rate. Due to the speed of the turnover and consistency of answers, we considered that the C-PAQ was easy to understand and complete by parents/legal guardians ( Supplementary Appendix 1 , online).

A scoping review was conducted in 84 studies, and the main determinants of children's physical activity during the pandemic were age, gender, socioeconomic background, and outdoor environment. 18 Guam et al. 19 demonstrated the concern about sedentary lifestyles in children caused by the COVID-19 pandemic and proposed solutions for physical activities at home. Dunton et al. 20 studied the early effects of the pandemic on physical activity and sedentary behavior in children living in the United States. They provided alerts for increased risk of obesity, diabetes, and cardiovascular disease in children and suggested that programmatic and policy strategies should be geared toward promoting physical activities and reducing sedentary behaviors over the following 12 months. 20 Moore et al. 21 described the immediate collateral consequences of the COVID-19 outbreak, which demonstrated an adverse impact on the movement and play behaviors of Canadian children (5–17-years-old). The results indicated that families spent less time on physical activities and spent more time on sedentary behaviors. Some parents have reported adopting new hobbies or accessing new resources. 21 We found a decrease in physical activity and an increase in sedentary activity, which was similar to previous studies. However, after using C-PAQ-PT in our study, we were able to quantify the changes in the intensity and frequency of physical and sedentary activities.

The quantification of changes caused by social distancing in activities was considered a strong point of our study. We have also identified some weaknesses, such as the potential limitations of a survey conducted through the internet. 22 Although subjective or self-reported measures have proven to be a promising means of gathering information, they do have their limitations. 23 As compared with other studies, the number of children studied was relatively small. 24 25 26 Another factor that may have influenced the results was that all questionnaires were answered by parents with some relationship with the researchers.

Regarding gender, Sá et al. 24 indicated that differences between sexes among 816 children were found with regards to screen play time (boys > girls) and playing without physical activity (girls > boys). In our study, we did not find significant differences, except in two activities, where we observed a decrease in the practice of tag and cycling among girls and boys, respectively.

Siegle et al. 8 described a decrease in the levels of physical activity, with no difference between the three age groups (3–5, 6–9, and 10–12 years) among Brazilian children during social isolation. In our study, the 10 to 14-year-old group had significantly greater participation in sports than the 5 to 9-year-old group. This difference may be related to the activity in physical education classes at school. Meanwhile, the group of 5 to 9-years-old can carry out activities in smaller spaces, with fewer children.

When analyzing the daily activities of children during restriction according to the type of house, Zagalaz-Sánchez et al. 26 found that the time dedicated to physical activity of those who live in a house with a garden and in apartments with more than 121 m ^2^ was greater than that of those living in flats less than 60 m ^2^ . Pombo et al. 27 confirmed that children with outdoor spaces in the household were significantly more active. We were unable to reproduce these results in our study, and did not find a statistically significant difference between residents of different types of housing.

A systematic review and meta-analysis performed by Pfledderer et al. 28 provided evidence that disparities in physical activity and health outcomes existed between urban and rural youth. Another study demonstrated that the presence of a park with free space for physical activity near the local residence was associated with a lower BMI among adults. 29 Based on these studies, we believed that the children included in our study who lived in smaller cities (< 500,000 inhabitants) would be less impacted than children who live in big ones (> 500,000 inhabitants); however, no significant differences were found.

The social withdrawal due to the COVID-19 pandemic has caused a significant reduction in activities that can be performed outdoors, in groups, or related to school activity in children and adolescents. In addition to having fewer, less-demanding activities performed indoors, this may be a significant indicator of a reduction in the level of physical activity in children during the period of social isolation induced by COVID-19, which may have an impact on orthopedic practice. Raitio et al. 30 observed a significant decrease in surgical procedures due to injuries related to daily, school, and sports activities performed by children during the period of social isolation. In an Italian trauma center, 31 the number of pediatric patients decreased by 84.6% in this period; there were no observed cases of trauma related to injuries at school or high-energy lesions. A sedentary lifestyle in children is associated with obesity, which in turn is associated with orthopedic complications during growth and development.

As for the impact on sports medicine, recovery efforts will be necessary to ensure the survival and threats of youth sports in the future.

Physical activities in the elderly are important for reducing physiological changes taking place on the locomotor apparatus. 32 For these reasons, the authors believe that future studies should include both children and the elderly.

## Conclusion

The use of C-PAQ-PT allowed a quantitative assessment to identify there was a reduction in the time for physical activities and an increase in the time for sedentary behaviors. During the week and on weekends, children and teenagers in Brazil are more isolated than they were before the pandemic.

Furthermore, only two physical activities were influenced by gender. The changes occurred in physical activities and sedentary behaviors, which were more common for each age group. The number of inhabitants in the city and type of housing did not influence changes.

## Supplementary Material

Anexo Suplementar 1 Supplementary Appendix 1
