# Supplementary material for: The Impact of COVID-19 Social Isolation on Physical Activity and Sedentary Behavior in Brazilian Children and Adolescents
Source: Rev Bras Ortop (Sao Paulo). 2025 Mar 12;60(1):s00441800941. doi: 10.1055/s-0044-1800941 (PMC11903117; doi:10.1055/s-0044-1800941)
Supplement: Supplementary file 1 — Anexo Suplementar 1 [file 10-1055-s-0044-1800941-s2400177pt.pdf]

CHILDREN'S PHYSICAL ACTIVITY QUESTIONNAIRE (C-PAQ)

Versão traduzida e adaptada culturalmente para o português do Brasil(C-PAQ.BR)

Questionário para os pais ou responsáveis

Nome da criança ou adolescente:_____________________________________

Data de nascimento da criança ou adolescente:_____/_____/_____

Assinale sua relação com a criança: ( )mãe / ( )pai / ( ) responsável / ( )outros

*Observações: Você levará aproximadamente 10 minutos para responder esse questionário

- Por favor, responda às perguntas em relação à criança mencionada acima.
- Por favor responda **TODAS** as pergunta do questionário, não deixe nenhuma sem responder.

Para informações adicionais, favor contatar:

**Seu filho praticou as seguintes atividades?**		Segunda a Sexta		Sábado e Domingo	
		**Quantas vezes na semana**	**Total de horas ou minutos**	**Quantas vezes por semana**	**Total de horas ou minutos**
**Exemplo: Andar de bicicleta**	Sim Não	**02**	**40 min**	**1**	**15min**
**ATIVIDADES ESPORTIVAS**					
Aeróbica	Sim Não				
Base 4	Sim Não				
Basquete/Volei	Sim Não				
Queimada	Sim Não				
Dança	Sim Não				
Futebol	Sim Não				
Ginástica	Sim Não				
Handebol	Sim Não				
Lutas(todas)	Sim Não				
rouba bandeira	Sim Não				
Jogo de Taco	Sim Não				
Corrida	Sim Não				
Aulas de natação	Sim Não				

Seu filho praticou as seguintes atividades?		Segunda a Sexta		Sábado e Domingo	
		Quantas vezes na semana	Total de horas ou minutos	Quantas vezes por semana	Total de horas ou minutos
Natação por diversão	Sim Não				
Tênis	Sim Não				
**ATIVIDADES DE LAZER**					
Andar de bicicleta (exceto à escola)	Sim Não				
Empinar pipa	Sim Não				
Boliche	Sim Não				
Tarefas domesticas	Sim Não				
Brincar de "casinha"	Sim Não				
Brincar no parquinho (play-grounds)	Sim Não				
Brincar com animais estimação	Sim Não				
Patins	Sim Não				
Patinete	Sim Não				
Skate	Sim Não				
Polícia e ladrão	Sim Não				
Pular corda	Sim Não				
Pega Pega(Todos os tipos)	Sim Não				
Caminhar com o cachorro	Sim Não				
Caminhada/trilha	Sim Não				
**ATIVIDADES NA ESCOLA**					
Aula de Educação Física	Sim Não				
Ir à escola a pé<br>(ida e volta = 2 vezes)	Sim Não				
Ir à escola de bicicleta<br>(ida e volta = 2 vezes)	Sim Não				
Outros? se sim, Favor relatar:	Sim Não				
**OUTRAS ATIVIDADES**					
Arte e artesanato (ex: cerâmica ou costura)	Sim Não				
Desenho, pintura	Sim Não				
Lição de casa	Sim Não				
Jogos faz de conta	Sim Não				
Escutar música	Sim Não				
Brincar com brinquedos dentro de casa	Sim Não				
Jogos de tabuleiro/cartas	Sim Não				

**Seu filho praticou as seguintes atividades?**		**Segunda a Sexta**		**Sábado e Domingo**	
		**Quantas vezes na semana**	**Total de horas ou minutos**	**Quantas vezes por semana**	**Total de horas ou minutos**
Jogos de computador/eletrônicos/celular/tablet	Sim Não				
Tocar instrumento musical	Sim Não				
Leitura	Sim Não				
Sentar e conversar	Sim Não				
Falar ao telefone	Sim Não				
Ida e volta à escola de carro ou ônibus	Sim Não				
Navegar na internet	Sim Não				
Assistir TV/vídeos	Sim Não				
Outros? se sim, Favor relatar:	Sim Não				
