# Supplementary material for: The Impact of COVID-19 Social Isolation on Physical Activity and Sedentary Behavior in Brazilian Children and Adolescents
Source: Rev Bras Ortop (Sao Paulo). 2025 Mar 12;60(1):s00441800941. doi: 10.1055/s-0044-1800941 (PMC11903117; doi:10.1055/s-0044-1800941)
Supplement: Related texts

# Research article: O impacto do isolamento social da Covid-19 na atividade física e no comportamento sedentário de crianças e adolescentes brasileiros

**Keywords:** adolescente, Covid-19, criança, exercício físico, inquéritos e questionários

## Abstract

**Objetivo** Este estudo mediu o impacto do isolamento social nas atividades físicas e no comportamento sedentário de crianças e adolescentes brasileiros e avaliou a influência de fatores como idade, sexo, dias da semana, tipo de moradia e população da cidade.

**Métodos** O estudo foi aprovado pelo Comitê de Ética Médica e o consentimento dos pais dos participantes foi obtido. Um *Google Form* (Google LLC, Mountain View, CA, USA) foi enviado aos pais por *e-mail* e WhatsApp (Meta Platforms Inc., Menlo Park, CA, USA). Os pais responderam às perguntas do *Children's Physical Activity Questionnaire* em sua versão em português (C-PAQ.PT) duas vezes, um mês antes e durante o isolamento social.

**Resultados** Houve redução estatisticamente significativa na média de atividade física ( *p* < 0,001) e aumento na média de atividades sedentárias ( *p* < 0,001) nos dias úteis e finais de semana durante o isolamento social. Houve maior redução em basquete, handebol e corrida no grupo de 10 a 14 anos, assim como em brincar em parquinhos no grupo de 5 a 9 anos. Houve diminuição estatisticamente significativa na prática de pega-pega e ciclismo, respectivamente, entre meninas e meninos. Não observamos diferenças estatisticamente significativas entre os tipos de moradia e o número de habitantes na cidade.

**Conclusão** O C-PAQ.PT permitiu uma avaliação quantitativa para identificação das variações nas atividades físicas e comportamentos sedentários durante o isolamento social. Observamos que apenas duas atividades físicas foram impactadas pelo sexo dos participantes. As mudanças não foram influenciadas pelo número de habitantes da cidade ou pelo tipo de moradia.

## Introdução

Em 30 de janeiro de 2020 e 11 de março de 2020, respectivamente, a Organização Mundial da Saúde declarou a infecção por *coronavirus disease 2019* (Covid-19) uma emergência global de saúde pública e pandemia. Muitos países implementaram uma série de medidas de controle como forma de preparo e resposta à Covid-19. Bloqueios completos ou parciais foram instituídos para evitar a disseminação da doença. Medidas sociais, incluindo restrições de movimentação, fechamento de escolas e empresas, isolamento social e restrições de viagens internacionais, foram implementadas para evitar a introdução e limitar o movimento do vírus. 1

Uma forma de avaliar o nível e a frequência de atividade física em crianças fora do ambiente escolar utiliza acelerômetros, que funcionam como sensores de movimento. Este é um método que permite coletar informações de forma imediata, com baixo custo e grande aplicabilidade, considerando diferentes contextos e práticas de atividade física. 2 Em uma situação de isolamento social, o uso de um acelerômetro para esta pesquisa era praticamente impossível. Por esse motivo, realizamos uma busca bibliográfica para identificar métodos alternativos que pudessem permitir a avaliação do grau e da frequência de atividade física entre crianças fora do ambiente escolar sem comprometer o isolamento social. Questionários de autorrelato ainda podem ser a única maneira viável de avaliação da atividade física em muitas situações e aspectos que não são facilmente mensurados de maneira objetiva. 3 Questionários foram propostos para diferentes faixas etárias e validados pela comparação de questionários com o uso concomitante de acelerômetros. 4 5 6

Embora a proliferação de estudos epidemiológicos baseados na web, empregando técnicas de recrutamento *online* , tenha aumento, a abordagem ideal para maximizar as taxas de recrutamento permanece incerta. 7

Apesar da existência de estudos realizados no Brasil 8 e revisões sistemáticas, 9 10 há poucas avaliações quantitativas do impacto do distanciamento social na atividade física e no comportamento sedentário em crianças. O objetivo primário deste estudo foi identificar e quantificar se as atividades físicas e o comportamento sedentário sofreram modificações em comparação aos níveis pré-pandêmicos em crianças e adolescentes durante o período de isolamento social. Os objetivos secundários foram investigar se as mudanças foram influenciadas por idade, sexo, dias úteis ou finais de semana, tipo de moradia (vertical ou horizontal) e número de habitantes da cidade.

## Materiais e Métodos

O estudo observacional transversal foi aprovado pelo Comitê de Ética Médica sob o número CAAE: 31168920.6.0000.5505. Foi necessária a participação de voluntários, como pais ou responsáveis, que tivessem relações pessoais com os pesquisadores e respondessem às perguntas. Um termo de consentimento informado foi incluído antes do questionário para informar a todos os pais ou responsáveis que eles consentiriam em participar da pesquisa ao clicar no item “Quero participar”. Foram incluídas respostas de pais ou responsáveis que moram com crianças de ambos os sexos, com idades entre 5 e 14 anos, em uma cidade e passaram por isolamento social devido à pandemia de Covid-19. Foram incluídas respostas de pais ou responsáveis que moram com crianças de ambos os sexos. As respostas aos questionários foram excluídas caso repetidas.

## Formulário

O Google Forms (Google LLC., Mountain View, CA, EUA) foi usado para desenvolver o questionário específico para o propósito desta investigação. Empregamos a tradução e adaptação transcultural do *Children's Physical Activity Questionnaire* (C-PAQ) 11 12 para o português. 13 O formulário tinha 49 perguntas sobre atividades físicas e comportamentos sedentários. As respostas foram diretas e o questionário pôde ser concluído em poucos minutos. Os pais ou responsáveis foram convidados a participar da pesquisa em duas ocasiões. A primeira foi baseada nas atividades realizadas por crianças e adolescentes cerca de um mês antes do isolamento social. A segunda foi feita com base nas atividades realizadas por crianças e adolescentes ao receberem o formulário durante o isolamento social. Além das respostas do questionário, também solicitamos informações sobre os dados de identificação da criança, a cidade e o tipo de moradia. A pesquisa foi distribuída a 350 pais ou responsáveis por meio de *e-mail* e WhatsApp (Meta Platforms Inc., Menlo Park, CA, USA) entre 1° de junho de 2020 e 15 de junho de 2020.

Para realização da análise, as crianças foram divididas em dois tipos de moradia, sendo eles apartamento (vertical) ou casa (horizontal), e cidades com mais ou menos de 500.000 habitantes. As duas faixas etárias, de 5 a 9 anos e 10 a 14 anos, foram escolhidas por serem as mais comuns entre os estudantes brasileiros do ensino fundamental e médio.

## Metodologia Estatística

Usamos o teste de Wilcoxon para avaliar as diferenças entre antes e durante o isolamento. O teste qui-quadrado foi usado para determinar se as variáveis eram dependentes ou associadas entre si. Cada comparação foi associada a um valor de *p* . Esta análise estatística utilizou the IBM SPSS Statistics for Windows (IBM Corp., Armonk, NY, EUA) version 20.0, Minitab 16 (Minitab Inc. State College, PA, EUA) e Microsoft Excel 2010 (Microsoft Corp., Redmond, WA, EUA).

## Resultados

Dentre os 171 formulários preenchidos, 35 foram excluídos por falta de informações que impossibilitaram a análise dos dados. No total, 136 formulários foram analisados. Em relação à idade, os grupos de crianças de 5 a 9 anos e de 10 a 14 anos tinham 70 e 66 participantes, respectivamente. Os grupos compostos por apartamentos e casas foram compostos por 52 e 84 crianças, respectivamente. Em relação ao gênero, 66 crianças eram do sexo feminino, 65 do sexo masculino e 5 não foram identificadas pelos pais. Recebemos questionários de moradores de 27 cidades, distribuídas em oito estados do Brasil. Em relação ao porte da cidade, 102 crianças residiam em cidades com mais de 500 mil habitantes, 32 com menos de 500 mil habitantes e duas crianças não informaram onde residiam.

A Fig. 1 mostra as 45 atividades que tiveram variação no número de crianças e adolescentes na comparação antes e durante o isolamento social. Destas, 17 e 28 foram consideradas atividades físicas e sedentárias, respectivamente. Houve redução significativa no número de praticantes, tempo total e frequência de atividades como basquete, queimada, futebol, handebol, lutas, pega-bandeira, corrida, aulas de natação, natação por diversão, andar de bicicleta, brincar no parquinho, pega-pega, aula de educação física, ir a pé para a escola e ir de carro ou ônibus para a escola ( Fig. 2 ). As atividades realizadas em ambientes fechados tiveram um aumento significativo em tempo total e na frequência apesar da ausência de aumento significativo no número de participantes em atividades de desenho e pintura, uso de brinquedos em ambientes fechados, jogos de tabuleiro/cartas, jogos em dispositivos eletrônicos, sentar-se e conversar, navegar na internet e assistir a vídeos/televisão ( Fig. 3 ). Algumas atividades foram praticadas apenas por algumas crianças, o que resultou em um alto grau de incerteza nas estimativas.

**Fig. 1:** O gráfico retrata o número de crianças e adolescentes envolvidos em cada atividade antes e durante o isolamento social.

**Fig. 2:** O gráfico indica a taxa de redução do tempo gasto e da frequência de cada atividade.

**Fig. 3:** O gráfico indica um aumento no tempo gasto e na frequência de cada atividade.

A frequência e a duração de cada atividade foram categorizadas para determinar o número de minutos de prática de cada atividade ao longo dos dias úteis e fins de semana, antes e durante a pandemia. A categorização de seu comportamento foi baseada na frequência média (número de vezes por semana) e intensidade média (tempo), como pode-se ver na Tabela 1 . Obtivemos o tempo médio praticado pelos participantes em todas as atividades multiplicando frequência por intensidade. Comparamos, portanto, o tempo gasto praticando atividades antes e durante a pandemia em dias úteis e fins de semana. Para esta análise, consideramos apenas as respostas dos participantes que citaram a prática tanto antes quanto durante a pandemia. Dessa forma, tivemos dados pareados, ou seja, quando o mesmo participante é o pesquisador e seu controle.

**Tabela 1:** Comportamentos categorizados por frequência (número de vezes por semana) e intensidade (tempo)

Frequência	Categorização
1–2 vezes	1,5
3–4 vezes	3,5
5 ou mais vezes	5
**Intensidade**	
1–30 minutos	15
31–60 minutos	45
61–180 minutos	120
Mais de 180 minutos	240

Durante o isolamento social, houve redução na duração da atividade física e aumento dos comportamentos sedentários, tanto nos dias úteis quanto nos fins de semana. Durante os dias úteis, a média de atividade física antes e durante o isolamento social foi de 197,0 e 153,4 minutos, respectivamente ( *p* < 0,001). Durante os fins de semana, a média de atividade física antes e durante o isolamento social foi de 192,1 e 149,4 minutos, respectivamente ( *p* < 0,001). Durante os dias úteis, a média de atividades sedentárias antes e durante o isolamento social foi de 268,1 e 393,3 minutos, respectivamente ( *p* < 0,001); e nos fins de semana, de 251,8 e 346,2 minutos, respectivamente ( *p* < 0,001), como mostrado na Tabela 2 .

**Tabela 2:** Comparação da duração da atividade física antes e durante a pandemia (teste de Wilcoxon)

Isolamento social			Média	Mediana	DP	Q1	Q3	N	IC	Valor de *p*
Atividade física	Semana	Antes	197,0	67,5	262,4	22,5	180,0	273	31,1	< 0,001
		Durante	153,4	52,5	270,5	22,5	157,5	273	32,1	
	Fim de semana	Antes	192,1	67,5	304,9	22,5	180,0	177	44,9	0,019
		Durante	149,9	22,5	282,2	22,5	157,5	177	41,6	
Sedentarismo	Semana	Antes	268,1	157,5	352,5	22,5	420,0	1.378	18,6	< 0,001
		Durante	393,3	157,5	414,6	52,5	600,0	1.378	21,9	
	Fim de semana	Antes	251,8	67,5	354,4	22,5	420,0	1.143	20,5	< 0,001
		Durante	346,2	157,5	412,6	22,5	600,0	1.143	23,9	

**Abreviações:** DP, desvio padrão; Q1, primeiro quartil; Q3, terceiro quartil; IC, intervalo de confiança.

No isolamento social, houve redução nas atividades que podem ser realizadas ao ar livre em grupo ou relacionadas às atividades escolares e aumento na frequência e quantidade de participação em atividades sedentárias em ambientes fechados. No grupo de 10 a 14 anos, houve maior redução no basquete, handebol e corrida; no grupo de 5 a 9 anos, houve maior redução nas brincadeiras no parquinho. Houve diminuição estatisticamente significativa nas práticas de pega-pega e ciclismo entre meninas e meninos, respectivamente. Na maioria das atividades, não houve diferenças estatisticamente significativas entre os diferentes tipos de moradia. Quanto à frequência, apenas as atividades de uso de brinquedos em ambientes fechados e assistir televisão/vídeos apresentaram diferença entre os grupos. Embora a diferença na redução dessas atividades tenha sido estatisticamente significativa, seus valores são pequenos por serem atividades com redução relativamente pequena na amostra como um todo. O número de habitantes da cidade não teve significância estatística, ou seja, não influenciou as mudanças nas atividades de crianças e adolescentes.

## Discussão

A diminuição da frequência e intensidade das atividades físicas e sedentárias entre crianças durante o isolamento social decorrente da pandemia da Covid-19 pode ter impacto no hábito de manutenção de uma vida saudável, incluindo o equilíbrio postural em crianças. 14

Questionários autorrelatados foram usados em larga escala para avaliação e monitoramento da atividade física em crianças e adolescentes. O C-PAQ é uma ferramenta que pode ser baixada do website ( https://www.mrc-epid.cam.ac.uk/wp-content/uploads/2014/08/CPAQ.pdf ), tendo sido previamente validado. 12 Por suas características, o consideramos uma boa ferramenta para avaliação do impacto do isolamento social devido à pandemia da Covid-19 nas atividades de crianças e adolescentes residentes no Brasil. A tradução e adaptação cultural de um questionário de autoavaliação permitiu a utilização de instrumentos para comparação de grupos com idiomas e culturas diferentes, o que foi muito útil na prática clínica. 15 16 17 As oito atividades trocadas para a cultura brasileira apresentaram alta taxa de resposta. Devido à rapidez da rotatividade e consistência das respostas, consideramos que o C-PAQ foi de fácil compreensão e preenchimento pelos pais/responsáveis legais ( Anexo Suplementar 1 , *online* ).

Em uma revisão de escopo de 84 estudos, os principais determinantes da atividade física das crianças durante a pandemia foram idade, sexo, origem socioeconômica e ambiente externo. 18 Guam et al. 19 demonstraram a preocupação com o sedentarismo infantil causado pela pandemia da Covid-19 e propuseram soluções para atividades físicas em casa. Dunton et al. 20 estudaram os primeiros efeitos da pandemia na atividade física e no comportamento sedentário em crianças que vivem nos Estados Unidos. Estes pesquisadores alertaram para o aumento do risco de obesidade, diabetes e doenças cardiovasculares em crianças, sugerindo que as estratégias programáticas e políticas deveriam ser voltadas para a promoção de atividades físicas e redução de comportamentos sedentários nos 12 meses seguintes. 20 Moore et al. 21 descreveram as consequências colaterais imediatas da pandemia de Covid-19, que demonstraram um impacto adverso na movimentação e nos comportamentos lúdicos em crianças canadenses (5–17 anos). Os resultados indicaram que as famílias gastaram menos tempo em atividades físicas e mais tempo em comportamentos sedentários. Alguns pais relataram adotar novos *hobbies* ou acessar novos recursos. 21 Encontramos uma diminuição na atividade física e um aumento na atividade sedentária, semelhante aos estudos anteriores. No entanto, após o uso do C-PAQ-PT, conseguimos quantificar as mudanças na intensidade e frequência das atividades físicas e sedentárias.

A quantificação das mudanças causadas pelo distanciamento social nas atividades foi considerada um ponto forte do nosso estudo. Também foram identificadas algumas fragilidades, como as possíveis limitações de uma pesquisa realizada pela internet. 22 Embora as medidas subjetivas ou autorrelatadas sejam comprovadamente um meio promissor de coleta de informação, têm suas limitações. 23 Em comparação a outros estudos, o número de crianças estudadas foi relativamente pequeno. 24 25 26 Outro fator que pode ter influenciado os resultados foi que todos os questionários foram respondidos por pais com algum relacionamento com os pesquisadores.

Sá et al. 24 indicaram que diferenças entre os sexos de 816 crianças foram relacionadas ao tempo de jogo de tela (meninos > meninas) e brincar sem atividade física (meninas > meninos). Em nosso estudo, não encontramos diferenças significativas, exceto em duas atividades, onde observamos uma diminuição na prática de pega-pega e ciclismo entre meninas e meninos, respectivamente.

Siegle et al. 8 descreveram uma diminuição nos níveis de atividade física, sem diferença entre as três faixas etárias (3–5, 6–9, 10–12 anos) entre as crianças brasileiras durante o isolamento social. Em nosso estudo, o grupo de 10 a 14 anos teve participação significativamente maior em esportes do que o grupo de 5 a 9 anos. Essa diferença pode estar relacionada à atividade nas aulas de educação física na escola. Enquanto isso, o grupo de 5 a 9 anos consegue fazer atividades em espaços menores, com menos crianças.

Ao analisar as atividades diárias das crianças durante o isolamento de acordo com o tipo de casa, Zagalaz-Sánchez et al. 26 constataram que o tempo dedicado à atividade física daqueles que moravam em casas com jardim e em apartamentos com mais de 121 m ^2^ era maior do que aqueles em apartamentos com menos de 60 m ^2^ . Pombo et al. 27 confirmaram que crianças com espaços ao ar livre no domicílio eram significativamente mais ativas. Não conseguimos reproduzir esses resultados em nosso estudo e não encontramos diferença estatisticamente significativa entre crianças de diferentes tipos de moradia.

Uma revisão sistemática e metanálise realizada por Pfledderer et al. 28 forneceu evidências de disparidades na atividade física e nos resultados de saúde entre jovens urbanos e rurais. Outro estudo demonstrou que a presença de um parque com espaço livre para atividade física perto da residência estava associada a um índice de massa corporal (IMC) mais baixo entre adultos. 29 Com base nesses estudos, acreditamos que as crianças incluídas em nosso estudo que viviam em cidades menores (< 500.000 habitantes) seriam menos impactadas do que as crianças que viviam nas grandes (> 500.000 habitantes); no entanto, não foram encontradas diferenças significativas.

O isolamento social devido à pandemia da Covid-19 causou uma redução significativa nas atividades que podem ser realizadas ao ar livre, em grupo ou relacionadas à atividade escolar em crianças e adolescentes. Além de haver menos atividades de menor exigência em ambientes fechados, isso pode ser um indicador significativo de redução no nível de atividade física em crianças durante o período de isolamento social induzido pela Covid-19, o que pode ter um impacto na prática ortopédica. Raitio et al. 30 observaram uma diminuição significativa nos procedimentos cirúrgicos devido a lesões relacionadas às atividades diárias, escolares e esportivas realizadas por crianças durante o período de isolamento social. Em um centro de trauma italiano, 31 o número de pacientes pediátricos diminuiu 84,6% neste período; não foram observados casos de traumas relacionados a lesões na escola ou lesões de alta energia. Um estilo de vida sedentário em crianças está associado à obesidade que, por sua vez, está relacionada a complicações ortopédicas durante o crescimento e desenvolvimento.

Quanto ao impacto na medicina esportiva, esforços de recuperação serão necessários para garantir a sobrevida e diminuir as ameaças aos esportes juvenis no futuro.

A atividade física em idosos é importante para reduzir as alterações fisiológicas que ocorrem no aparelho locomotor. 32 Por essas razões, os autores acreditam que estudos futuros devem incluir tanto crianças quanto idosos.

## Conclusão

O uso do C-PAQ-PT permitiu uma avaliação quantitativa para identificar a redução no tempo para atividades físicas e aumento no tempo para comportamentos sedentários. Durante a semana e nos finais de semana, crianças e adolescentes no Brasil ficaram mais isolados do que antes da pandemia.

Além disso, observou-se que apenas duas atividades físicas foram influenciadas pelo sexo. As mudanças ocorreram nas atividades físicas e nos comportamentos sedentários mais comuns para cada faixa etária. O número de habitantes na cidade e o tipo de moradia não influenciaram as mudanças.
